# The impact of health education interventions on HPV vaccination uptake, awareness, and acceptance among people under 30 years old in India: a literature review with systematic search

**DOI:** 10.3389/frph.2023.1151179

**Published:** 2023-05-05

**Authors:** Eleni Krokidi, Arathi P. Rao, Elena Ambrosino, Pierre P. M. Thomas

**Affiliations:** ^1^ Faculty of Health, Medicine and Life Sciences, Maastricht University, Maastricht, Netherlands; ^2^ Prasanna School of Public Health, Manipal Academy of Higher Education, Manipal, India; ^3^ Department of Health Policy, Prasanna School of Public Health, Manipal Academy of Higher Education, Manipal, India; ^4^ Institute for Public Health Genomics, Faculty of Health, Medicine and Life Sciences, Maastricht University, Maastricht, Netherlands

**Keywords:** HPV—human papillomavirus, health education, vaccines, cervical cancer, adolecent girls

## Abstract

**Background:**

The HPV vaccine is used as one of the main prevention tools for HPV-related cancers globally, yet it is not part of the Indian National Immunization program. In light of the introduction of the indigenous vaccine, we examine the effectiveness of health education about uptake, acceptance, and awareness.

**Methods:**

Research was performed in the following databases: PubMed, CINAHL, Scopus, and Embase to identify studies between 2008 and 2022. Studies were included if: they were conducted in India including primary data research and health education intervention, and participants were between 9 and 29 years old.

**Results:**

Out of the 10.952 results, 7 studies were included. Four studies focused on adolescent girls, aged from 9 to 20 years old, and 3 on university students aged from 17 to 26 years. Five studies were implemented in urban areas and 2 in rural areas. Health education interventions proved to be effective in increasing uptake, awareness, and acceptance of the HPV vaccine. The barriers included among others: cost, lack of awareness, and cultural barriers.

**Conclusion:**

Observations from this study outline immediate action for policymakers to educate and encourage the young population toward HPV vaccination. Future programs should be aimed at different population groups and be adjusted according to their special characteristics and needs. Attention should be given to the male population and marginalized groups. The involvement of various stakeholders proved to be beneficial, and it is highly recommended.

## Introduction

Infection with the Human Papillomavirus (HPV) is the most common sexually transmitted infection (STI) of the reproductive tract with the majority of the sexually active population estimated to acquire the virus at some point in their lifetime (1, 2). The HPV family consists of more than 150 viral genotypes 13 of which, are identified as carcinogenic or high risk (3). Persistent infections along with lifestyle factors are associated with more than 90% of cervical and anal cancers as well as a significant percentage of oropharyngeal, penile, vagina, andvulvar cancers (1). Notably, 90% of the new infections will clear up without long-lasting implications. Implementation of preventive vaccination against the carcinogenic genotypes has been shown to significantly reduce the incidence of HPV-related cancer cases. However, various limiting reasons such as high cost and vaccine hesitancy have curbed the efforts of worldwide adoption of the preventive HPV vaccine, which prompts further investigation into the subject (1, 4, 5).

Globally, it is estimated that 570,000 cancer cases in women and 60,000 cases in men per year are related to HPV. The majority of them occurring in low and middle income countries. More specifically, 275,429 and 29,324, almost half of the global HPV-related cancer cases, occurin Asia in women and men respectively (6, 7). In India, a country home to one-sixth of the global population, cervical cancer is the second most common women's cancer in the country (8). Moreover, it accounts for around 25% of the global burden of disease in mortality and morbidity (9) representing a major threat to women's health (10, 11). However, over the years women's health in India is facing social and cultural challenges. The Indian society is highly patriarchal leading to health inequalities for women. For example, the birth sex ratio in India has always been skewed towards men, leading to numerous issues in some parts of the country (12). Sexual relations are still shrouded in taboo and stigma in India, particularly among young and unmarried women. The overall shame associated with women's sexual activity is carried over to STIs, including HPV, which makes diagnosis even more challenging (13). Besides stigma, women in India often face discrimination, also from healthcare workers, when seeking help for sexual and reproductive health rights (SRHR) matters (14).

Numerous efforts have been undertaken in India to control cervical cancer. In 1975 the government of India launched the first National Cancer Control Program aiming to provide cytology-based exams for women in premier cancer institutions (15). In 2010, the program became part of the National Program for Prevention and Control of Cancer, Diabetes, Cardiovascular Diseases, and Stroke (NPCDCS). This program aimed to control non-communicable diseases by focusing on common lifestyle factors such as smoking, diet, and alcohol consumption (16). In 2016, a mobile technology platform for cervical cancer screening was introduced by the Indian Ministry of National and Family Welfare (MoNFW) to provide support and monitoring of the screening programs in each state (15). The preferred screening method is visual inspection methods such as visual inspection with acetic acid (VIA) over the Papanicolaou smear test (Pap-test) and HPV-DNA tests (17). The preference of VIA can be justified by its cost-effectiveness, the immediate availability of the results, and the difficulties of Pap and HPV-DNA test samples preservation. However, VIA has poor sensitivity especially during the early stages of cervical cancer, whereas the alternative PAP and HPV-DNA tests have been proven more sensitive in early stage diagnosis, resulting in better outcomes for the patient (17). Besides the multifarious efforts of the Indian government, the screening coverage in India remains poor and the need for universal cervical cancer screening is still largely unmet (15). The implications of the current system's inefficiency is reflected at the majority of cervical cancer cases are being diagnosed in an advanced stage with poor prospects of survival (18).

However, the development of HPV vaccines has offered new opportunities for early prevention. Approximately 80% of cancers caused by HPV can be prevented by administering the HPV vaccine (6). Through the Cervical Cancer Elimination Strategy, the World Health Organisation (WHO) and United Nations Children's Fund (UNICEF) aim to increase the HPV vaccination global coverage to 90% of adolescent girls by 2030 globally. This target that aligns with the Sustainable Development Goals (SDGs) agenda (target 3.b essential medicines and vaccines) (19). In India, two HPV vaccines were licensed in 2008: a quadrivalent vaccine (Gardasil ™) and a bivalent vaccine (Cervarix™). Both vaccines use virus-like particles (VLPs) which are formulated by HPV surface components. In case of infection, antibodies will coat the virus preventing it from releasing its genetic material (8). Both vaccines provide high protection for HPV-related cancers. More specifically, they provide approximately 90% protection from cervical cancer with the antibodies remaining stable for at least 10 years (20). HPV vaccines in India are still not part of the National Immunization program, they are only available under prescription privately from the age of 9 (21). HPV vaccination uptake in India is low due to its elevated cost (approximately INR 3,000 per dose), misinformation regarding safety and effectiveness, and discouraging cultural perceptions for vaccines (11). Vaccine acceptance has been low, particularly after issues regarding the ethical procedures of HPV vaccine administration (21).

Nevertheless, the landscape of availability and acceptance of the HPV vaccination in India is promising. Since 2016, cost-effective vaccination programs against HPV have been successfully implemented in different states, achieving high coverage (21). In July 2022 the Drugs Controller General of India (DCGI) authorized an indigenous quadrivalent vaccine developed by the Serum Institute of India (22). On September 1st, 2022, more details were announced. The new quadrivalent vaccine will be available for both females and males and the estimated cost is INR 200–400 per dose suggesting high coverage in the young population and affordability (21–23). According to recent news, the Indian government is likely to begin HPV vaccination campaigns in six states of India in July 2023. However, there is currently no official announcement available (24).

The role of health education appears central to the successful implementation of the HPV vaccination strategy, as shown by the problems due to misingormationthat arose during the first rollout of a vaccination program. Health education has been widely used to increase health literacy, especially among young people, helping them to adopt healthy habits in a variety of fields (25, 26). Health education approaches have been successfully implemented to increase HPV vaccination uptake and awareness globally (27) while age-adjusted education helps young people to comprehend the importance and the relevance of HPV vaccination (28). However, the conservative attitudes of the Indian society toward health education and specifically on topics related to SRHRs particularly among the Indian youth, raise issues curtailing its effectiveness (29).

To date, there is ample questionnaire-based evidence assessing the knowledge and the acceptance of the HPV vaccine among the youth in India. However, the existing research is solely based on extracting information regarding these sectors without applying any intervention to improve them. Moreover, the lack of systematic review on the impact of health education regarding uptake, knowledge, acceptance, and willingness to get vaccinated represents a significant gap of knowledge (30–33). Notably, in the light of the new, indigenous HPV vaccination, it is necessary to examine the most efficient form of health education to enhance HPV vaccination among young people in India. This systematic review aims to address the following question, “what is the influence of health education on HPV vaccination uptake, knowledge, acceptance, and willingness to get vaccinated among people under 30 years old in India?”. Our findings will be useful to policymakers, the Indian Ministries of Education and the MoNFW, and researchers.

## Methods

In this systematic review, we examine the effectiveness of health education among people from 9 years old, which is the minimum age to receive the HPV vaccination. Our upper limit is 29 years old as, 90% of the Indian population has their first sexual intercourse before the age of 30. The current vaccine guidelines suggest that the first dose should be administrated before the first sexual contact (34).In June 2022 we conducted a pilot research followed by a systematic literature research in July 2022 on the following databases: PubMed, CINAHL, Embase, and Scopus. As featured in Table 1, the research string was not limited to titles and abstracts. The articles included were written in English between 2008, when the HPV vaccine was first licensed in India, and July 13th, 2022 articles for which abstracts and full text were not found, as well as articles not reporting any health education intervention were excluded. The research protocol adhered to the Preferred Reporting Items for Systematic Reviews and Meta-Analyses (PRISMA) 2020 guidelines (35). The keywords, the results, the filters used, the inclusion and the exclusion criteria are summarized in the Table 1.

**Table 1:** Databases, filters, keywords, results, inclusion and exclusion criteria.

Database	Filters	Results
PubMed	Language: English, Species: Humans, Date: 2008- 13/07/2022, Text availability: Full text	938
Scopus	Language: English, Date: 2008-13/07/2022, Affiliation Country: India	7.195
CINAHL	Language: English, Date: 2008-13/07/2022	191
Embase	Language: English, Date: 2008-13/07/2022, Species: Humans	2.628
Total	–	10.952
Research string	(“ Human Papillomavirus vaccine*” OR “HPV Vaccine*” OR “Human Papilloma Virus Vaccine*” OR “Cervical Cancer vaccine*” OR HPV OR “Human Papillomavirus “ OR “Human Papilloma Virus “ OR “cervical cancer”) AND (India* NOT “American Indian*”) AND (“health education” OR “health promotion” OR “community health education “ OR “health literacy” OR prevention OR knowledge OR awareness OR willingness OR “health intervention"	
Inclusion criteria	❖ Qualitative, quantitative, and mixed methods studies ❖ Studies reporting primary data ❖ Published between 2008 and 13th July 2022 ❖ Age group 9–29 years ❖ English language ❖ Peer-reviewed journals ❖ Grey literature	
Exclusion criteria	❖ Non-Indian residents ❖ Participants diagnosed with an HPV-related cancer	

### Selection

Two researchers independently screened the results (titles and abstracts) resulting from the electronic searches and evaluated the full-text articles. Discrepancies were solved *via* discussion and with the aid of a third reviewer who has expertise in the sexual and reproductive health field. Qualitative, quantitative, and mixed methods studies reporting primary data in English language published in peer-reviewed journals or grey literature between 2008 and 13th July 2022 were included. Studies were excluded if: they reported primary data from non-Indian residents, the age group was outside the age limits of 9–29 years, and if the participants have already been diagnosed with an HPV attributable cancer.

## Results

**Figure 1:**
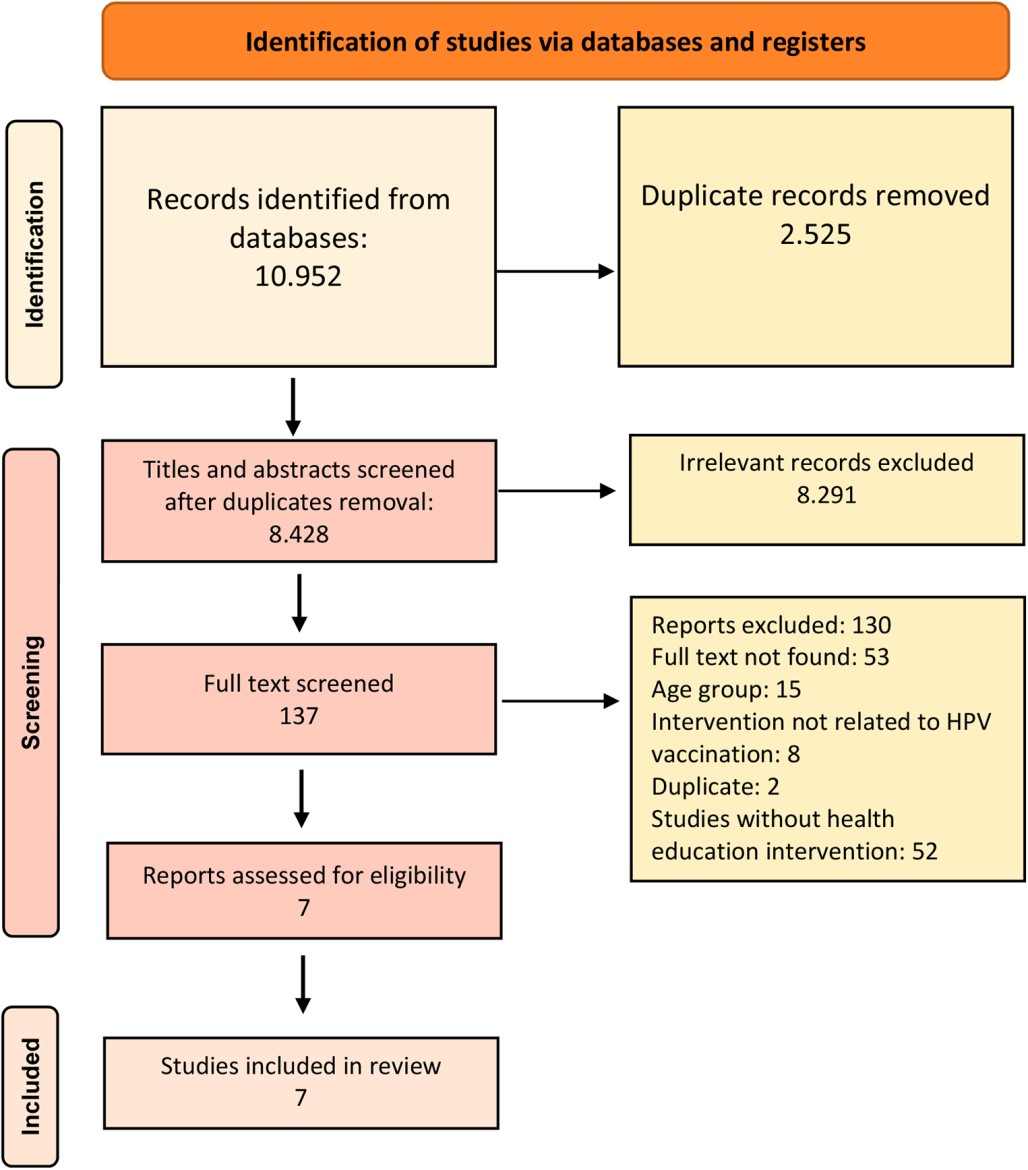
PRISMA 2020 flowchart.

The PRISMA flowchart (Figure 1) summarizes the review process. In total, 10.952 studies were screened and after the removal of duplicates and title screening 407 studies were eligible for abstract screening, 137 of which were eligible for full-text screening. After the full-text screening, 7 studies were included in our review with reasons of exclusion being: the absence of health education intervention, ineligiblility of the age group, unrelated intervention to HPV vaccination, geographical location, full text not found, duplicate (Table 2).

**Table 2:** Demographics.

Title	Experience of Human Papillomavirus Vaccination Project in a Community Set Up-An Indian Study	Acceptability of human papillomavirus vaccination among medical students in Mangalore, India	An exploratory study of undergraduate healthcare student perspectives regarding human papillomavirus and vaccine intent in India	Human papillomavirus (HPV) vaccine introduction in Sikkim state: Best practices from the first statewide multiple-age cohort HPV vaccine introduction in India-2018–2019	Preparedness of young girls for prevention of cervical cancer and strategy to introduce the HPV vaccine	Knowledge and awareness of HPV infection and vaccination among urban adolescents in India: A cross-sectional study	Effectiveness of Health Awareness Programme on Knowledge Regarding Cervical Cancer and Human Papilloma Vaccine among Adolescent Girls at Waghodia Taluka
Year	2021	2019	2021	2022	2018	2013	2019
Authors	Mandal et al.	Padmanabha et al.	Shetty et al.	Ahmed et al.	Swain & Parida	Ramavath & Olyai	Baria et al.
Journal	Asian Pacific Journal of Cancer Prevention	Elsevier Vaccine	SAGE Women's Health	Elsevier Vaccine	Indian Journal of Community Medicine	The Journal of Obstetrics and Gynecology in India	International Journal of Nursing Education
Type of Study	Not mentioned	Not mentioned	Cross-sectional observational study	Multi-staged cohort study	Quasi-experimental pretest and posttest design	Cross-sectional	Pre- experimental one group pre-test-post-test design
Location	West Bengal (Rural)	Kasturba Medical College, Mangalore, Karnataka	K S Hegde Medical Academy (KSHEMA), Mangalore, Karnataka	Schools in Sikkim state	College at Bhubaneswar, Odisha	Ahmedabad (Gujarat), Cuttack (Odisha), Lucknow Gwalior and Visakhapatnam	Waghodiya taluka village, Gujarat
Sample group	Girls and parents	Medical students	Undergraduate students in the healthcare department	Age-eligible girls: 199 and other stakeholders	College students (girls)	Adolescent girls	Adolescent girls
Size	555 girls	263 participants	20 students (11 males/9 females)	279 participants	60 participants	1000 participants	80 participants
Age	Girls 9–14 years	20–22 years	18–26 years	Girls 9–13 years/Age of other participants: unknown	17–24 years	13–19 years	9–20 years

### Demographics

**Figure 2:**
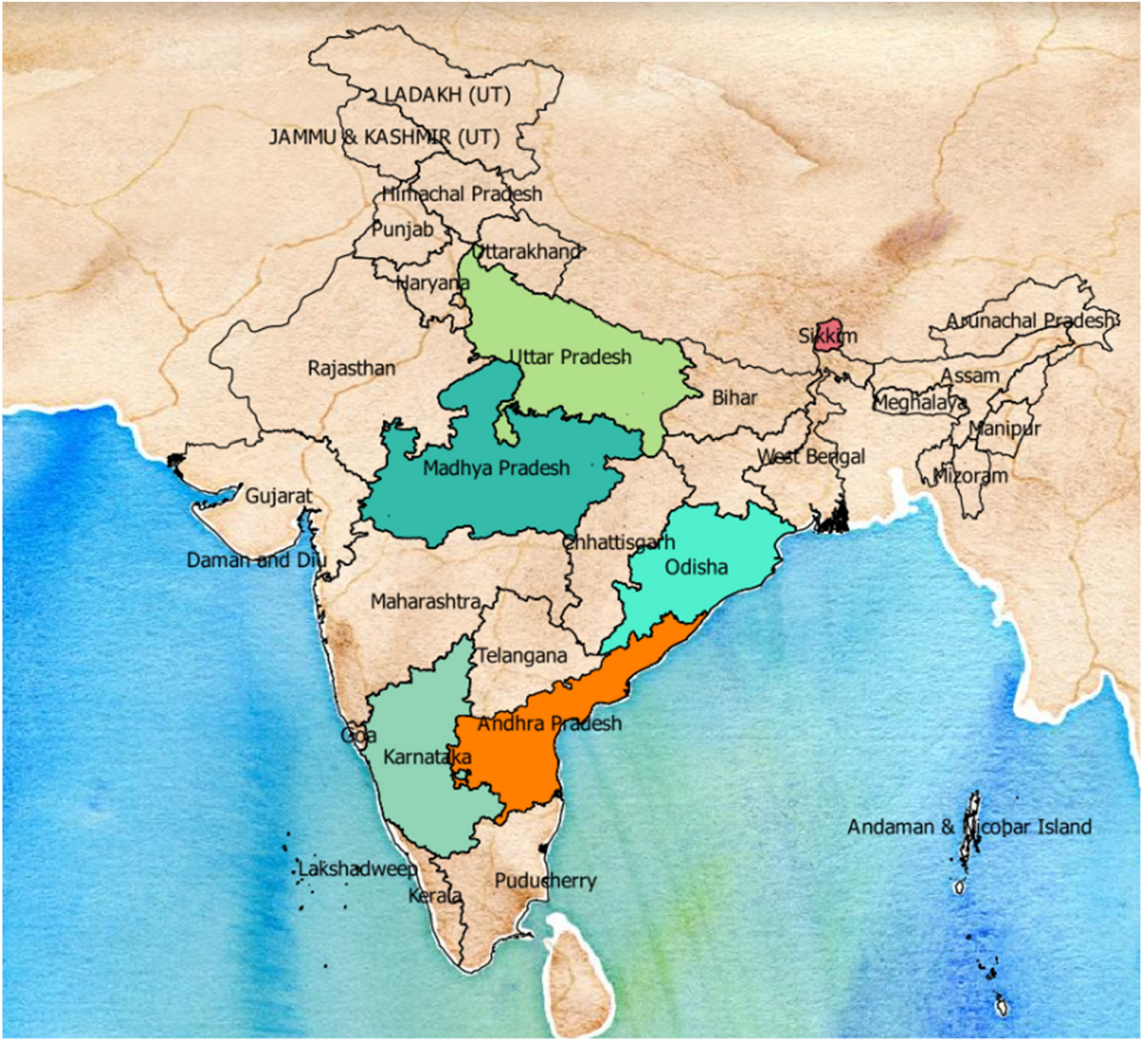
Study areas of health education for HPV in India. (Map creation program: QGIS).

Seven eligible studies based on the inclusion and exclusion criteria, with the earliest reported in 2013, were included in our systematic review (Table 2). Four studies focused on adolescent girls, aged from 9 to 20 years old, and 3 on university students aged from 17 to 26 years (2, 36–38). Out of these 7 studies, two included both female and male participants. Both studies were university-based studies and the participants were undergraduate healthcare students (39, 40). The geographical location of the studies varied, with 2 of them taking place in rural areas and 2 in universities in Mangalore (Figure 2) (39, 40).

### Types of health education and evaluation

Different types of health education interventions were reported yet some of the studies did not feature analytical descriptions of the methods (Tables 3, 4). The majority included audio-visual presentations and workshops. In the study by Mandal et. al., an orientation camp awareness session was held targeting adolescent girls and their parents and identified with the involvement of local stakeholders being identified as essential in encouraging the local community (36). Similarly, a community-based approach was employed by the state of Sikkim using several types of health education tools such as training, community-based workshops, and leaflets to target different stakeholders including adolescent girls (37). Two studies targeting healthcare students in Mangalore by Padmanabha et al. and by Shetty et. al reported the use of audio-visual presentations lasting from 5 to 20 min (39, 40). In a study by Ramavath & Olyai targeting adolescent girls in schools and colleges across urban India, the researchers provided lectures focusing on health (2). Colleges in Bhubaneswar implemented a 3-sessions sensitization program informing adolescent girls about the epidemiology of cervical cancer and prevention measures as well as about HPV vaccination (41). Lastly, in the study of Baria et al. taking place in Waghodiya Taluka village, Gujarat the health education method used was reported as a “health awareness program”, however no further details about the methods are mentioned (38).

**Table 3:** types of evaluation, health intervention and results.

Authors	Mandal et al.	Padmanabha et al.	Shetty et al.	Ahmed et al.	Swain & Parida	Ramavath & Olyai	Baria et al.
Type of evaluation of the health intervention	NA	Questionnaire regarding the knowledge about HPV/HPV vaccine and if students were vaccinated and why followed by the information session. After the intervention a second questionnaire regarding the acceptance and the barriers	Group discussions	Interviews	Pre-intervention and post-intervention questionnaires	Pre-intervention and post-intervention questionnaires	Pre-intervention and post-intervention questionnaires
Results	555 girls received 1st dose and 544 girls received their 2nd dose	After the intervention, about 60% of the participants answered positively about receiving the HPV vaccine, 7% denied and 34% were not sure.	Willingness to get vaccinated was higher among females than males with cost and cultural concerns being the main barriers. All participants were positive to promote the HPV vaccine in community settings and making it affordable.	More than 95% of the target population received both doses after health education. No severe side effects were reported. Factors that led to the successful vaccination campaign included among others strong political commitment.	Improvement in the knowledge of symptoms and prevention was identified after the intervention. 52 girls agreed to receive the vaccine however, only 35 of them were vaccinated.	Poor knowledge was reported among the participants with about 70% not being aware of cervical cancer and HPV as a cause of it. However, participants were willing to receive more knowledge and vaccination. The intervention significantly improved knowledge, awareness, and acceptance with 74.4% of the participants agreeing to receive the vaccine.	Before the intervention participants had inadequate knowledge of HPV vaccination and cervical cancer with the health education intervention improving significantly their knowledge.

**Table 4:** Type of evaluation prior to health intervention.

Authors	Mandal et al.	Padmanabha et al.	Shetty et al.	Ahmed et al.	Swain & Parida	Ramavath & Olyai	Baria et al.
Type of evaluation prior to health intervention	Prevaccine questionnaires	Questionnaire	N/A	N/A	Pre-intervention questionnaires	Pre-intervention questionnaires	Pre-intervention questionnaires

### Uptake

Out of 7 studies, the participants had the opportunity to receive the HPV vaccine following the health education intervention in only 3 studies. In the state of Sikkim and rural West Bengal, the uptake among adolescent girls was reported to be above 95%. In a study implemented in a college in Bhubaneswar although 86% of the girls agreed to receive the vaccine, only 58.33% received their first dose. In 3 studies it was reported that some of the participants had already taken the vaccine, but the percentage was low (3,33%, 10%, and 21% of the total participants) (36, 37, 41).

### Awareness

Four studies examined the awareness of the participants regarding HPV, cervical cancer, HPV vaccine, screening methods, and symptoms of cervical cancer two of which reported poor awareness (2, 38–40). In the study by Shetty et al. among 20 healthcare students in Mangalore, more than 50% of the participants were identified to have adequate knowledge with the majority of which were medical students. In the same study, it was demonstrated that HPV vaccine knowledge was relatively low among male participants in comparison to females (40). The study by Baria et al. in Waghodiya taluka village reported moderate to inadequate knowledge among adolescent participants before the intervention with significant improvement after the intervention (38).

Similarly, all the studies that reflected on the knowledge of the participants after the intervention reported an improvement in the participant's knowledge (2, 37, 38, 41).

### Acceptance and willingness

Four studies examined the acceptance and willingness to get vaccinated. Although acceptance and willingness appear distinct terms they were similarly used in the included studies (2, 39, 40, 41). In a study among healthcare students, all the females expressed their desire to get vaccinated, except the ones who were already vaccinated. However, in the same study, the male participants appeared to be hesitant even after the health education intervention (40). In the other 3 studies, the vaccination willingness varied from about 60% to 86% (2, 39, 41). Only one study examined if the participants proceed to vaccination (see uptake) (41).

### Barriers

The reasons underlying the HPV vaccination hesitancy were manifold (Table 2). The reported percentage express the percentage of participants who identified the mentioned reasons as barriers to the uptake of the HPV vaccine.

## Discussion

This review systematically collected reports from primary studies done in India on the effectiveness of health education interventions in improving the uptake, awareness, and acceptance of HPV vaccination among people aged 9–29 years and provides an evidence base for further research studies and policy. Our study's results highlight the effectiveness of health education in improving all the sectors that have been studied. Poor awareness was reported to be common prior to interventions, especially among the general population. In 3 studies where the participants were provided the opportunity for vaccination, the uptake was between 58,33% and more than 95%. Overall, female participants demonstrated greater acceptance and awareness compared to men. A variety of health education methods were identified. Additionally, our study provides information on the underlying causes of HPV vaccine hesitancy with cost and lack of awareness being the major reasons among others. Our results align with the results of the systematic review conducted by Thulaseedharan et al. in 2019 (42). Thulaseedharan et al. focused on awareness, attitude, and acceptance toward HPV/cervical cancer, screening, and HPV among Indians from 1993 to 2017 whereas our study additionally reflected on the health education interventions and their effectiveness (42).

### Future opportunities

The authorization of the indigenous HPV vaccine to enter the market in July paves the way for achieving the Cervical Cancer Elimination goal of 90% vaccination of girls up to 15 years old by 2030 (43). Furthermore, although India was greatly affected by the COVID-19 pandemic, only 63.3% are fully vaccinated against SARS-CoV-2, implying a greater vaccine hesitancy problem. Therefore, there is a need to increase vaccination coverage in both HPV and Covid-19 vaccines (44). With the Indian government taking measures to develop the country's economy, the country has the potential to increase immunization coverage by investing in healthcare (45). In these renewed efforts, health education should play an indispensable role as one of the main strategies targeting the young generation. To develop efficient health education and communication strategies, it is suggested that well-informed policymakers should advise communication guides developed by the WHO. For example, the “HPV vaccine communication guide” released in 2016 includes basic communication advice, information about the safety of the HPV vaccine, and how to build a strategy for different population groups. These tools provide significant aid when developing vaccine communication strategies in India which host diverse population groups (46).

Furthermore, policymakers could consider best practices from successful vaccination efforts implemented in India and overseas. Our research highlights the successful approach of the state of Sikkim where more than 95% of the target population received both doses (41). The success is multifactorial such as the detailed implementation strategy and the involvement of several stakeholders such as religious and political leaders, doctors, and parents. Moreover, this HPV vaccine introduction program included a variety of different educational materials pending on the target group. The program included an evaluation strategy with more than 200 interviews among the stakeholders and parents and observations in community areas. Similar methods were used in Punjab where an additional HPV cost-effectiveness analysis was conducted. The results of this analysis demonstrated the HPV vaccine was cost-effective in this state by reducing lifetime risk for cervical cancer by 64%. Moreover, the role of adolescent-friendly clinics (AFCs), an intervention under Reproductive, Maternal, New-born, Child, and Adolescent Health (RMNCH + A) is important. Among their SRH services, AFCs could add the provision of counselling, awareness, and HPV vaccines (47).

### Health education strategies in south Asia and the US

Our research identified a lack of HPV vaccine health education-related studies conducted in neighbouring countries such as Sri Lanka, Nepal, and Bangladesh which highlights the need for global evidence-based strategies in South Asia (48). However, there is a plethora of strategies conducted in high-income countries. Rani et al. who conducted a systematic review on public education interventions in the United States of America (USA) in 2020, identified 30 eligible studies (49). The USA is a high-income country with a 756 million population and the HPV-vaccine uptake is approximately 50% among adolescents. In this study, communication strategies delivered by experts and they were addressed to both parents and young adolescents resulted in increased uptake (49). Another systematic review in the USA was implemented on practice- and community-level interventions. The authors recommended that future programs should be implemented in communities such as schools or within healthcare settings such as counselling by physicians or reminder calls (50). In Greece, interactive presentations in schools had a positive effect on knowledge and acceptance of the vaccine among students (51). On the other hand in China, the most populous country in the world with low HPV-vaccination uptake rates, a web-based intervention proved to be effective during the COVID-19 pandemic (52). The target population was college girls and the intervention included 10 min of daily web-based education covering a variety of topics related to HPV vaccination and cervical cancer. The method used proved to be efficient since it improved both awareness and acceptance as well as affordable and easy to be applied, especially during lockdowns when gatherings are limited (52). It is recommended that future programs are inspired by the interventions presented in our research and hence involve several stakeholders and use a variety of educational methods according to the population group (e.g., parents, students), the settings (e.g., community-based), and the limitations of the program (e.g., during a pandemic).

### Reflection on the study population

Considering the findings of our study in which the main population group constists of students, HPV vaccine information is essential to be part of the curriculum of health education in schools. Moreover, since awareness appeared to be inadequate among university students health education and opportunistic vaccination could be part of the university's curriculum by organizing workshops, thematic days, etc (53). However, according to the Indian demographics, students are not the main population group in the country. As stated by a report of the National Commission on Protection of Child Rights (NCPCR) in 2013, only about 40% of the youth aged 15–18 attended school (54). We found 3 studies among university students when only around 27% of young Indians (age 18–23 years) were enrolled in higher education in 2017–18 (55). In these studies, healthcare, students demonstrated higher awareness. This is justified by their field of studies, and it should not be generalized to the majority of the Indian population. Moreover, only two studies including the males were identified in our research (39, 40). Males demonstrated poor awareness and acceptance compared to female participants. Additionally, 70% of Indians reside in rural areas whereas we found only 2 out of 7 studies that implemented in such locations (56). School attendance in rural areas (37%) is significantly lower than in urban areas (51%) among people between 15 and 18 years (54). Our research indicates that 5 out of 7 studies failed to provide adequate information about the health education methods that were used. A major gap both in research among gender and marginalised young population groups (illiterate, rural areas residents) and in the application of health education interventions was identified. To achieve high vaccination coverage in India, health education and youth interventions should be made accessible to the whole population in India. Thus, different methods and tools ought to be used considering the characteristics of the individual target population such as age, sex, socioeconomic status, area of residence, cultural and religious background, level of education, access to technology, etc. Since the indigenous vaccine will be available to both males and females more research and educational interventions is deemed necessary. Lastly, future researchers should elaborate on transparency regarding the tools and the methods used to increase visibility and reproducibility.

### Amelioration of existing strategies

The HPV vaccination strategies could be integrated into already existing screening efforts for cervical cancer prevention in India. Despite the existing guidelines, screening programs are mostly based on opportunistic screening or after the appearance of symptoms (17). The current screening programs target sexually active, married women between 30 and 65 years old (17). Nevertheless, the National Family Health Survey in 2015–2016 indicated that the mean age of first sexual intercourse among women is 19.1 years while 2% of the unmarried women participants (age 15–24 years) declared having sex before marriage. In the same survey, it was reported that only 22,3% of the eligible women were screened for cervical cancer between 2015 and 2016 with the screening percentage being higher among literate women (34). Thus, the current screening programs exclude a significant number of eligible women. Focusing on married women, it is evident that sexually active, unmarried women are side-lined. The main barriers to cervical cancer screening as reported by Thulaseedharan et al. do not differ significantly from the barriers reported in our study about HPV vaccinaton (Tables 5, 6) (42). On the other hand, anal cancer screening strategies are currently inadequate and little research has been done in this field. Studies and screening opportunities are limited to HIV-positive patients; the majority of which are male sex workers. The stigma surrounding sexual orientation and sex work in India often discourages patients from seeking help for their SRHR complaints, hence perpetuating the burden (57–59).

**Table 5:** HPV vaccination barries.

Barriers	Percentage of participants	Number of studies	Analytical studies
Cost of the vaccine	56.7%–100%	4	Padmanabha et al, 2019: 63% Shetty, 2021: 100% Swain and Parida, 2018: 81.3% Ramavath & Olyai, 2013: 56.7%
Safety of the vaccine	10%–15.6%	2	Padmanabha et al, 2019: 10% Ramavath & Olyai, 2013: 15.6%
Lack of awareness	10%–24.8%	3	Padmanabha et al, 2019: 10% Swain & Parida, 2018: 15,51% Ramavath & Olyai, 2013: 24.8%
Not perceiving themselves in danger/Not being sexually active	10%–81.3%	2	Padmanabha et al, 2019: 10% Swain & Parida, 2018: 81.3%
Side effects	8%–15.6%	2	Padmanabha et al, 2019: 8% Ramavath & Olyai, 2013: 15.6%
Cultural issues	55%	1	Shetty, 2021: 55%
Unwillingness of parents	22.41%	1	Swain & Parida, 2018: 22.41%
Fear of pain/needles	2.9%–17.24%	2	Swain & Parida, 2018: 17.24% Ramavath & Olyai, 2013: 2.9%

**Table 6:** HPV vaccination and screening barriers.

HPV vaccination barriers (current research)	Cervical cancer creening barriers (Thulaseedharan et al. 2019)
Cost of the vaccine	Lack of awareness (e.g. of cervical cancer screening procedure)
Safety of the vaccine	Lack of resources (e.g. time, money)
Lack of awareness	Provider-related barriers (e.g. discomfort with the pelvic examination by male providers)
Not perceiving themselves in danger/Not being sexually active	Not perceiving themselves in need (e.g. too old/too young)
Side effects	
Cultural issues	
Unwillingness of parents	Psychosocial (e.g. not approved by husband/relatives)
Fear of pain/needles	

Therefore, to achieve the goals of cervical cancer elimination strategy for 90% HPV vaccination coverage and 70% screening by 2030 there is an urgent need for amelioration and a combination of existing strategies (6). Future strategies should aim to tackle the screening and vaccination barriers by creating awareness through health education adjusted to the target population. Screening programs should extend their age limits including sexually active people regardless of their sex, marital status and sexual orientation. The scope and reach of current screening opportunities should be extended, providing screening for anal and other types of HPV-related cancers. However, since premarital sex, sexual orientation and sex work are still a taboo in India such intervention might not be feasible. HPV vaccination should tackle both males and females and awareness should be adjusted according to sex and cultural norms (13).

In rural areas, where cancer prevalence is alarming due to a variety of factors such as illiteracy, poor hygiene, and early marriage the role of Accredited Social Health Activist (ASHA) workers could be expanded by adding HPV vaccination counselling (17). ASHA workers are well-respected women in rural communities who work as the link between the public health system and the community (60). In 2016, the Rural Health Mission (RHM) accredited ASHAs as health mobilizers for cervical cancer screening. However, research by Khanna et al. in the Varanasi district identified that although ASHAs had good knowledge of cervical cancer, less than 10% of them were screened (61). Therefore, before extending the role of ASHAs, it is crucial to apply strategies for narrowing the gap between knowledge and practice. In this case, health education is essential for two reasons: to provide knowledge to ASHA workers regarding HPV vaccines and screening and to help them improve their health promotion strategies. It is evident that supporting ASHA workers and extending their role could lead to positive outcomes in terms of screening and vaccination (61).

Lastly, efforts out to be made to increase the vaccination rates among girls who do not attend school. In 2021, Holroyd et al. published research on designing a pro-equity HPV vaccine delivery program for girls who dropped out of school in a community set-up in India (62). Their findings highlight the necessity of the parents' involvement and education and the importance of out-of-school accessibility. Their recommendations include among others: the utilization of media to spread awareness to low-literacy populations, the engagement of vaccinated girls, and the adjustment of the vaccination programs to the needs of the target populations. Policy-makers should consider the results of such research to design health education interventions for minority populations in rural India (62).

### Limitations

The main limitation of our study lies in the inclusion of a diverse body of literature including qualitative, quantitative and mixed-methods studies. There was therefore no scope for the conduction of a meta-analysis on the present data due to the lack of consistent measures of outcomes and large heterogeneity of the studies included. In addition, the true effect of health education on HPV uptake in India may not be ascertained based on the findings of the current paper, due to the lack of studies based on experimental and randomized control trials. These study designs may have helped understanding the true effect of intervention. Furthermore, the results of this present paper may be generalised to India as a whole. In fact, the number of the studies included remains relatively small and the samples not representative. Lastly, the lack of official statistics and surveillance obtained from government sources (e.g., regarding the HPV vaccine uptake) and the absence of previous studies in this area is a matter of consideration.

## Conclusion

Considering the development of the indigenous HPV vaccine, India has the capacity to achieve success in the Cervical Cancer Elimination goal for 90% of coverage among girls up to 15 years old (6). With the introduction of the indigenous vaccine, the cost barrier being addressed. There is a need to consider the other barriers illustrated in our research such as the lack of awareness and the cultural barriers. Our research demonstrated that health education is an effective tool to improve awareness, uptake, and acceptance among the Indian young population. Proper-designed educational interventions, such as the one in Sikkim, could lead to an uptake of more than 95%. The results of our study demonstrated a gap in research among males and marginalized population groups such as girls who dropped out of school and people living in rural areas. Therefore, it is critical that future health education interventions are inclusive and accessible to the whole population in India. Policy-makers ought to take into account the individual characteristics of each target group and adjust communication interventions accordingly. The involvement of several stakeholders such as religious and political leaders proved to play a key role in the success of HPV vaccination strategies. Hence, it is recommended further programs include a variety of stakeholders as part of their communication strategy.
